# Purification and Characterization of an Extracellular, Thermo-Alkali-Stable, Metal Tolerant Laccase from *Bacillus tequilensis* SN4

**DOI:** 10.1371/journal.pone.0096951

**Published:** 2014-05-28

**Authors:** Sonica Sondhi, Prince Sharma, Shilpa Saini, Neena Puri, Naveen Gupta

**Affiliations:** 1 Department of Microbiology, BMS Block, Panjab University, Chandigarh, India; 2 Department of Industrial Microbiology, Guru Nanak Khalsa College, Yamunanagar, Haryana, India; Center for Nanosciences and Nanotechnology, Mexico

## Abstract

A novel extracellular thermo-alkali-stable laccase from *Bacillus tequilensis* SN4 (SN4LAC) was purified to homogeneity. The laccase was a monomeric protein of molecular weight 32 KDa. UV-visible spectrum and peptide mass fingerprinting results showed that SN4LAC is a multicopper oxidase. Laccase was active in broad range of phenolic and non-phenolic substrates. Catalytic efficiency (*k*_cat_/*K*_m_) showed that 2, 6-dimethoxyphenol was most efficiently oxidized by the enzyme. The enzyme was inhibited by conventional inhibitors of laccase like sodium azide, cysteine, dithiothreitol and β-mercaptoethanol. SN4LAC was found to be highly thermostable, having temperature optimum at 85°C and could retain more than 80% activity at 70°C for 24 h. The optimum pH of activity for 2, 6-dimethoxyphenol, 2, 2′-azino bis[3-ethylbenzthiazoline-6-sulfonate], syringaldazine and guaiacol was 8.0, 5.5, 6.5 and 8.0 respectively. Enzyme was alkali-stable as it retained more than 75% activity at pH 9.0 for 24 h. Activity of the enzyme was significantly enhanced by Cu^2+^, Co^2+^, SDS and CTAB, while it was stable in the presence of halides, most of the other metal ions and surfactants. The extracellular nature and stability of SN4LAC in extreme conditions such as high temperature, pH, heavy metals, halides and detergents makes it a highly suitable candidate for biotechnological and industrial applications.

## Introduction

Laccases (benzenediol: oxygen oxidoreductases; EC 1.10.3.2) are multicopper oxidases (MCOs) which catalyze the oxidation of a wide variety of organic and inorganic compounds with concomitant four electron reduction of molecular oxygen to water. They catalyze the oxidation of both phenolic and non-phenolic substrates. In general, laccases oxidize phenols and aromatic amines such as methoxyphenols, phenols, polyphenols, anilines, aryl diamines, hydroxyindols, benzenethiols and some cyanide complexes of metals. Laccases are very useful enzymes with respect to their applications in industry. They have found use in industrial and biotechnological applications such as in biobleaching, xenobiotics bioremediation, textile dyes decolorization, biosensors, food industry etc [1].

Laccases are widely distributed in nature. They have been found in almost all spheres of life but have been most extensively studied in fungi including *Ascomycetes, Basidiomycetes* and *Deuteromycetes* [2]. Fungal laccases are not stable in extreme conditions like temperature, pH, salt etc. which exists in industry. Moreover, the production of fungal laccases in large quantity is problematic due to the accumulation of large amount of fungal biomass. Bacterial laccases have several significant properties which are not characteristics of fungal laccases like stability at high temperature and pH [3], salt tolerance [4] etc. Only a few bacterial laccases have been characterized till date but they could not be exploited on an industrial scale as most of them are intracellular or spore bound [5]. There are some reports of extracellular laccase from *Sterptomyces* [6], [7], [8], [9] which are difficult to produce in large quantity because of the problems associated with their filamentous growth [10], slow growth rate and expensive downstream processing [11], thus limiting their applications. Therefore, there is a need to study laccases which are thermo-alkali-stable and produced extracellularly by bacteria. In this regard, previously we have isolated *Bacillus tequilensis* SN4 from the activated sludge of paper mill effluent treatment plant which produces a laccase extracellularly in the culture supernatant. Moreover, on preliminary analysis, this laccase was found to be highly thermostable (optima at 80–90°C) and alkali-stable which makes this laccase a potential candidate for application in industry [12]. In this study we have purified and characterized the enzyme with respect to properties which are important for its industrial applications.

## Materials and Methods

### Chemicals

Guaiacol, 2, 2′-azino bis[3-ethylbenzthiazoline-6-sulfonate] (ABTS), syringaldazine (SGZ), sephadex G-150 and DEAE-cellulose were purchased from Sigma (USA). Other substrates *viz.* pyrogallol, α-naphthol, p-phenylenediamine (PPD), resorcinol, 2, 6-dimethoxyphenol (DMP), catechol, 3,4-dihydroxy-phenylalanine (L-DOPA) and tyrosine were purchased from Hi-media. Other chemicals used were of analytical grade.

### Microorganism and growth conditions

The bacterial strain used in this study, *Bacillus tequilensis* SN4 MTCC no. 11828 (GenBank accession no. *KF150708*) producing extracellular thermo-alkali-stable laccase was previously isolated in our laboratory from activated sludge of paper mill effluent treatment plant [12].

### Laccase production

Laccase was produced in M162 medium containing [13], 0.6% yeast extract, 0.2% tryptone, 100 µM CuSO_4_ and 300 µM MnSO_4_. The medium was inoculated with 0.3% of 24 h old culture of *B. tequilensis* SN4. Flasks were kept at 30°C; 150 rpm agitation rate for 96 h. After incubation, the culture was centrifuged at 7826 x g for 15 min. The supernatant was used as extracellular enzyme.

### Laccase Assay

The enzyme assay was performed at 85°C using 2 mM DMP as substrate in 0.1 M Tris-HCl buffer (pH 8.0) for 5 min. The change in absorbance due to oxidation of DMP was monitored at 470 nm (ε = 14800 M^−1^ cm^−1^). One unit of laccase was defined as the amount of the enzyme required to transform 1 µmol substrate per min under standard assay conditions. Specific activity was calculated as U mg^−1^ of protein.

### Protein estimation

Protein concentration was determined by the method of Lowry et al. [14]. Bovine serum albumin was used as the standard. The proteins eluted from column chromatography were monitored by taking absorbance at 280 nm.

### Purification of SN4 laccase

The laccase from *B. tequilensis* SN4 was purified to homogeneity by using a combination of purification techniques. In the first step, proteins were precipitated with acetone [15]. Chilled acetone (60%) was added to the crude enzyme, kept for 2–3 h at −20°C and then centrifuged at 7826 x g for 10 min at 4°C. The pellet was kept in open to evaporate residual acetone and dissolved in 0.1 M Tris-HCl buffer (pH 8.0). The acetone precipitated proteins were applied to Sephadex G-150 (40×1.5 cm^2^) column pre-equilibrated with 0.1 M Tris-HCl buffer (pH 8.0). The protein was eluted with the same buffer at a flow rate of 1.0 ml min^−1^. The active fractions were pooled and concentrated by polyethylene glycol (PEG) [16] and then applied onto DEAE-cellulose column (15×1.2 cm^2^) pre-equilibrated with 0.1 M Tris-HCl buffer (pH 8.0). The bound proteins were eluted with a linear gradient of NaCl (0–1.0 M) at a flow rate of 0.8 ml min^−1^. The active fractions were pooled and concentrated through PEG and dialyzed against same buffer to remove NaCl. The purity of the enzyme was determined by running SDS-PAGE gel electrophoresis.

### SDS-PAGE and Activity Staining of Laccase Enzyme

To determine the purity of the protein and the molecular weight of laccase from *B. tequilensis* SN4, SDS- PAGE was performed under complete denaturing conditions [17]. Protein samples were heated for 5 min in the presence of SDS and β-mercaptoethanol. Electrophoresis was done with 5% stacking and 14% separating gel, stained with Coomassie Brilliant Blue R-250 dye and destained with methanol: acetic acid: distilled water (4∶1∶5). However, for activity staining, samples were heated for 5 min in the presence of SDS without β-mercaptoethanol (because β-mercaptoethanol inhibited the SN4 laccase activity).The staining of the gel was done with 2 mM guaiacol in 0.1 M Tris-HCl buffer (pH 8.0) at 60°C for half an hour. Laccase activity band was indicated by the development of reddish-brown color. Standard molecular weight protein markers (14–97 KDa) were used to calculate the molecular weight of laccase.

### Absorbance spectrum and MALDI-TOF analysis of SN4 Laccase

The UV-visible spectrum of the purified laccase was determined in 0.1 M Tris-HCl buffer (pH 8.0) from 200–800 nm. Identification of protein by peptide mass fingerprinting was carried out by MALDI-TOF/TOF analysis of purified protein. The band corresponding to laccase activity was excised from coomassie stained SDS-PAGE gel, digested with trypsin and peptides were extracted by the method of Shevchenko et al. [18]. For subsequent peptide spectra acquisition and analysis, the matrix-assisted laser desorption/ionization-time of flight (MALDI-TOF) was performed using AB SCIEX MALDI-TOF/TOF 5800. Mass spectrometry data were compared with database in the NCBI and Swiss Prot databases using the Mascot search algorithm.

### Substrate specificity and Kinetic Characteristics of SN4 laccase

The ability of SN4 laccase to oxidize several phenolic and non-phenolic substrates *viz.* ABTS, SGZ, guaiacol, pyrogallol, α-naphthol, PPD, resorcinol, DMP, catechol, L-DOPA and tyrosine was determined, at different pH values ranging from 1.0–10.0 [1.0–2.0 (0.1 M KCl-HCl buffer), 2.5–3.5 (0.1 M glycine-HCl buffer), 4.0–5.5 (0.1 M acetate buffer), 6.0–7.5 (0.1 M phosphate buffer), 8.0–9.0 (0.1 M tris-HCl buffer) and 9.5–10.0 (0.1 M carbonate-bicarbonate buffer)]. The relative rate of oxidation for each substrate (at their optimum pH) was compared using the enzyme activity with DMP as 100%.

For analyzing the kinetic properties of laccase, three conventional substrates oxidized by SN4 laccase *viz.* guaiacol, DMP and ABTS were taken at concentration of 100 to 5000 µM. Michaelis-Menton coefficient (*K*_m_) were determined by plotting Line-Weaver Burk plot for each substrate. *K*_cat_ and *V*_max_ were also calculated for each substrate.

### Effect of inhibitors on laccase activity

The effect of known inhibitors of laccase *viz.* sodium azide (NaN_3_), ethylenediaminetetraacetic acid (EDTA), diethylenetriaminepentaacetic acid (DTPA), cysteine monohydrate, dithiothreitol (DTT) and β-mercaptoethanol were studied at 1, 5 and 10 mM concentrations. The enzyme was incubated with the inhibitors for half an hour at 37°C with constant shaking at 150 rpm. The residual activity was then analyzed as per standard assay conditions.

### Effect of temperature on laccase activity and stability

Effect of temperature on purified laccase was determined by oxidation of DMP at temperature ranging from 55–100°C at an interval of 5°C. The maximum enzyme activity was taken as 100% and relative activities were plotted.

Thermostability of the enzyme was measured over the temperature range of 65–85°C by incubating the enzyme in thin-wall test tubes for a time period of 0–24 h. At different time intervals, aliquots were withdrawn and residual activity was determined under standard assay conditions.

### Effect of pH on laccase activity and stability

The pH dependence of laccase activity was determined for guaiacol (2 mM), ABTS (2 mM), DMP (2 mM) and SGZ (50 µM) as substrate by performing the enzyme assay at different pH values ranging from 1.0, 1.5 and 2.0 (0.1 M KCl-HCl buffer), 2.5, 3.0 and 3.5 (0.1 M Glycine-HCl buffer), 4.0, 4.5, 5.0 and 5.5 (0.1 M Acetate buffer), 6.0, 6.5, 7.0 and 7.5 (0.1 M phosphate buffer), 8.0, 8.5 and 9.0 (0.1 M Tris-HCl buffer) and 9.5 and 10.0 (0.1 M carbonate-bicarbonate buffer). The reaction with guaiacol, ABTS and DMP was carried out for 5 min and SGZ for 1 min. The oxidation of substrates was monitored at 465 nm for guaiacol (ε = 12000 M^−1^ cm^−1^), 420 nm for ABTS (ε = 36000 M^−1^ cm^−1^), 470 nm for DMP (ε = 14800 M^−1^ cm^−1^), and 525 nm for SGZ (ε = 64000 M^−1^ cm^−1^). The maximum enzyme activity was taken as 100% and relative activities were plotted.

Stability of the enzyme was measured at pH 8.0, 8.5 and 9.0 by incubating the enzyme in buffers of various pH values for 0–24 h at 65°C. At various time intervals, aliquots of enzyme were withdrawn and the residual activity was determined as per standard assay conditions.

### Effect of metal ions, halides and surfactants on laccase activity

The effect of halides, metal ions and surfactants on laccase activity was studied by preincubating the enzyme for 30 min at 37°C, 150 rpm with 0.5–1.0 mM conc. of various metal ions including FeSO_4_.7H_2_O, CuSO_4_.5H_2_O, NiSO_4_.6H_2_O, LiSO_4_.H_2_O, CaSO_4_.2H_2_O, CoSO_4_.7H_2_O, MnSO_4_.H_2_O, HgSO_4_, ZnSO_4_.7H_2_O, MgSO_4_.7H_2_O and Al_2_(SO_4_)_3_.16H_2_O; 100–500 mM conc. of various halides NaF, NaCl, NaBr, NaI and 0.1–1.0 mM concentration of surfactants including non-ionic (triton X-100, tween-20, tween-80), anionic (SDS), cationic (CTAB). Enzyme without any agent was taken as control. The residual activity was then analyzed as per standard assay conditions.

### Statistical Analysis

All the experiments were carried out in triplicates and the mean ± standard deviation has been plotted. Data was analyzed using analysis of variance (ANOVA) by Sigma Stat version 2.03 and values which were statistically significant (p value <0.05) were taken.

## Results

### Protein Purification and Molecular weight determination

**Figure 1:**
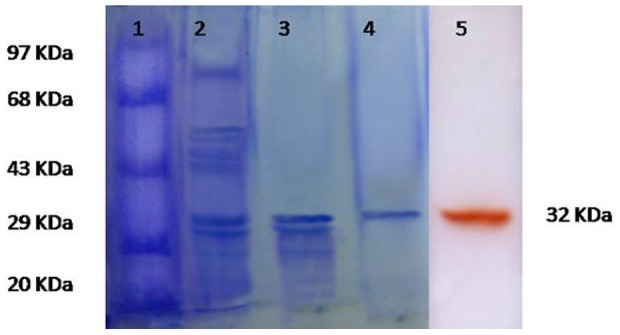
SDS-PAGE analysis of SN4LAC from *B. tequilensis* SN4. (Protein samples were denatured by heating for 5 min in the presence of SDS and β-mercaptoethanol): Lane 1: Protein markers, Lane 2: Acetone precipitated proteins, Lane 3: Sephadex-150 Column purified enzyme, lane 4: DEAE-Cellulose anion exchange Column purified enzyme, Lane 5: Activity staining; purified laccase stained with guaiacol (samples were heated for 5 min in the presence of SDS without β-mercaptoethanol).

Extracellular secretion of SN4 laccase made its purification easier in comparison to all other known bacterial laccases which are either intracellular or spore bound. The purification was carried out using a combination of routine chromatography procedures. After precipitation with 60% acetone; the protein was applied on Sephadex G-150 column; laccase active fractions were pooled and subjected to DEAE-cellulose column and eluted with NaCl (Figure S1 in File S1). After final step, the enzyme was purified to 28.46 fold with a yield of 13.34% (Table 1). The SDS-PAGE analysis of purified protein showed a band of 32 KDa which corresponded to the activity staining band of laccase (Fig. 1). This purified laccase was designated as SN4LAC.

**Table 1:** Summary of purification procedures for *B. tequilensis* SN4 laccase.

Purification steps	Total activity (U)	Total protein (mg)	Specific activity (U/mg)	Yield (%)	Purification fold
Crude (Culture supernatant)	5678.94	540	10.52	100	1
Acetone precipitation (60%)	3599.28	195	18.46	63.38	1.75
Sephadex G-150	1507.52	15.79	95.47	26.55	9.07
DEAE-cellulose	757.49	2.53	299.40	13.34	28.46

### UV-visible spectrum and MALDI-TOF analysis of SN4LAC

**Figure 2:**
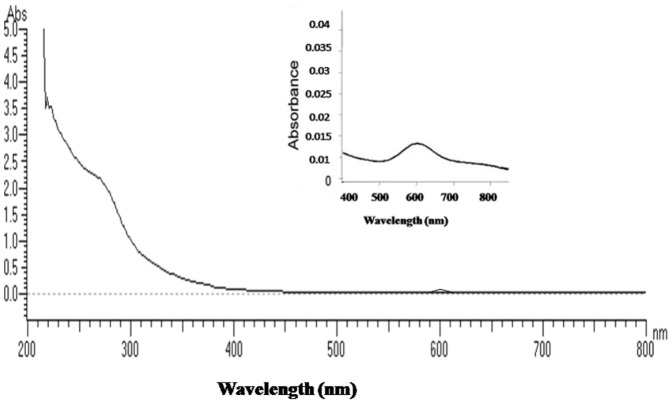
UV-visible spectrum (200–700 nm) of purified SN4LAC. The inset figure shows the zoomed image of the spectrum of SN4LAC in the range of 400–800 nm.

**Figure 3:**
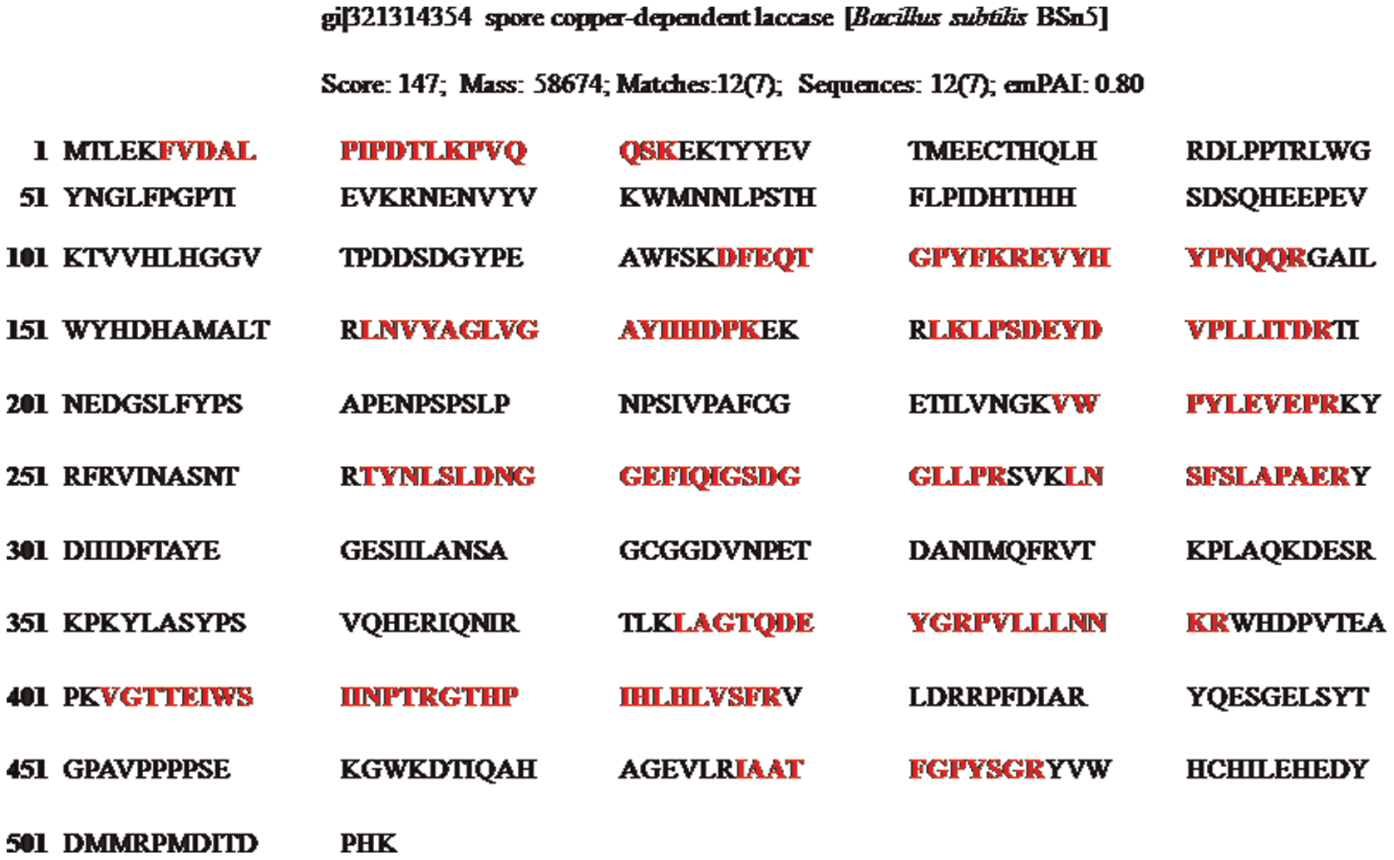
Peptide sequence showing identity with spore bound copper dependent laccase of *Bacillus subtilis* BSn5. Peptide sequences detected by tryptic digestion of SN4 laccase was shown in bold red.

UV-visible spectrum of purified SN4LAC showed an absorption peak at 600 nm (corresponding to the T1 copper centre) and a slight shoulder at 330 nm (corresponding to the T3 binuclear copper centre) (Fig. 2). On MS/MS analysis, the protein was identified with significant protein scores (p>0.05) from Mascot searches of peptide mass fingerprints (Figure S2 in File S2). On MALDI-TOF analysis of 32 KDa protein band showed homology with laccases of other strains of *B. subtilis* being maximum with spore bound copper dependent laccase from *B. subtilis* BSn5 [19] with a score of 147 and query coverage of 34% (Fig. 3).

### Substrate specificity and Kinetic properties of SN4LAC

SN4LAC was able to oxidize phenolic as well as non phenolic substrates. The optimum pH for each substrate was calculated and the rate of oxidation for different substrates was compared at their respective pH optima. SN4LAC oxidized o-phenols (in order of DMP>guaiacol>L-DOPA>pyrogallol>catechol), p-phenols (PPD) and other substrates of laccase such as ABTS, α-naphthol and SGZ, but poorly oxidized m-phenols (resorcinol) and no activity towards tyrosine was observed (Table 2).

**Table 2:** Substrate profile of Laccase from *B. tequilensis* SN4.

Substrate	ε (M^−1^ cm^−1^)	λ_max_ (nm)	Optimum pH	Relative activity (%)
DMP	14800	468	8.0	100
ABTS	36000	420	5.5	93.90±1.56
SGZ	64000	525	6.5	95.82±0.78
Guaiacol	12000	465	8.0	88.36±0.54
α-Naphthol	2200	330	6.0	93.95±1.25
Catechol	2211	450	7.0	19.21±1.38
Pyrogallol	35000	450	6.5	35.79±2.78
Tyrosine	12000	278	-	ND
PPD	14685	450	7.0	51.67±1.45
Resorcinol	6220	340	-	ND
L-DOPA	3600	475	9.0	69.17±0.24

ND- not detected.

Values represent mean ± SD (n = 3).

The reaction rate of SN4LAC was dependent on substrate concentration and followed Michaelis-Menton kinetics. The *K*_m_ and *k*_cat_ for ABTS was 80±4 µM and 291±2.7 s^−1^, for DMP was 680±27 µM and 11±0.1 s^−1^ and for guaiacol was 3.289±0.06 and 63±0.1 (Table 3).

**Table 3:** Kinetic properties of purified SN4LAC in comparison to other laccases.

Substrates	*Bacillus tequilensis* SN4			*Bacillus* sp. HR03 [23]			*Streptomyces ipomea* [8]		
	*K*_m_ (mM)	*k*_cat_ (s^−1^)	*k*_cat_/*K*_m_ (mM^−1^ s^−1^)	*K*_m_ (mM)	*k*_cat_ (s^−1^)	*k*_cat_/*K*_m_ (mM^−1^ s^−1^)	*K*_m_ (mM)	*k*_cat_ (s^−1^)	*k*_cat_/*K*_m_ (mM^−1^ s^−1^)
ABTS	1.404±0.08	67.04	47.82	0.535	127	237	0.4	9.99	24.975
DMP	0.840±0.012	73.15	87.05	0.053	3	56.60	4.2	4.20	1
Guaiacol	3.258±0.096	62.96	19.34	-	-	-	-	-	-

Values represent mean ± SD (n = 3).

### Effect of inhibitors on laccase activity

SN4LAC was inhibited by the common inhibitors of laccase (Table 4). When reducing agents *viz.* cysteine, DTT and β-mercaptoethanol were added, no enzyme activity could be observed. 10 mM concentration of NaN_3_, EDTA and DTPA decreased the enzyme activity to 32%, 22.8% and 23.62% respectively.

**Table 4:** Effect of inhibitors on SN4LAC activity.

Inhibitors	Conc. (mM)	Relative activity (%)
Control	----	100
Sodium azide	1	76.95±3.82
	5	53.86±1.40
	10	32.57±1.22
DTPA	1	33.99±.66
	5	31.82±0.72
	10	23.81±1.06
EDTA	1	42.45±1.03
	5	27.83±0.18
	10	21.53±1.18
Cysteine	1	63.75±1.65
	5	1.84±0.05
	10	1.45±0.05
Dithiothreitol	1	20.56±1.28
	5	0
	10	0
B-mercaptoethanol	1	0
	5	0
	10	0

Values represent mean ± SD (n = 3) relative to untreated control sample.

### Temperature and pH optima and stability

**Figure 4:**
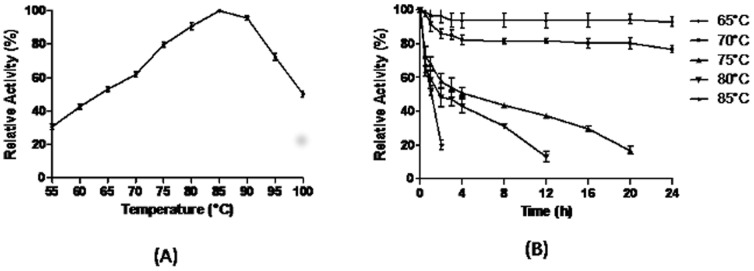
Effect of temperature on SN4LAC activity. (a) Optimum temperature of laccase activity (b) Stability of enzyme at various temperatures.

The purified SN4LAC showed maximum activity in the temperature range of 80–90°C having optima at 85°C (Fig. 4a). The enzyme could retain 50% activity even at 100°C. The SN4LAC retained more than 80% activity at 70°C and was completely stable at 65°C for 24 h. The half life of SN4LAC was 4 h, 3 h and 1 h at 75°C, 80°C and 85°C respectively (Fig. 4b).

**Figure 5:**
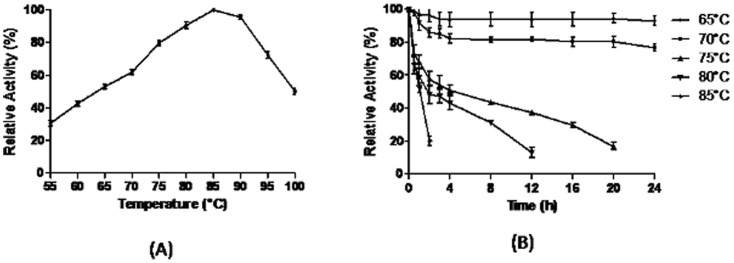
Effect of pH on SN4LAC activity. (a) Optimum pH for different substrates (b) Stability of laccase at different pH values.

The pH optima of purified SN4LAC for four different substrates *viz.* ABTS, DMP, SGZ and guaiacol was found to be 5.5, 8.0, 6.5 and 8.0 respectively (Fig. 5a). Enzyme was found to be highly stable in the alkaline pH range. The enzyme could retain 75% activity even after 24 h incubation at pH 9.0 (Fig. 5b).

### Effect of halides and metal ions on SN4LAC activity

SN4LAC retained 75–80% activity at 500 mM concentration of halides. Cu^2+^ and Co^2+^ increased the SN4LAC activity to 126% and 150% respectively at 5.0 mM concentration and in the presence of other metal ions, enzyme retained 70–80% activity. Hg^2+^and Fe^2+^ inhibited the laccase activity to 27% and 40% respectively (Table 5).

**Table 5:** Effect of metal ions and halides on SN4LAC activity.

Metal Ions	Conc. (mM)	Relative Activity (%)
Control	-	100
CuSO_4_.5H_2_O	1.0	106.56±1.36
	5.0	126.09±1.54
CaSO_4_.2H_2_O	1.0	83.66±1.58
	5.0	75.47±0.92
NiSO_4_.6H_2_O	1.0	86.25±1.85
	5.0	76.84±1.58
HgSO_4_	1.0	51.99±1.47
	5.0	26.60±1.22
MgSO_4_.7H_2_O	1.0	99.18±0.70
	5.0	76.99±1.22
MnSO_4_.H_2_O	1.0	83.59±1.10
	5.0	79.48±0.66
LiSO_4_.H_2_O	1.0	82.38±1.51
	5.0	77.81±0.84
CoSO_4_.7H_2_O	1.0	117.98±2.50
	5.0	150.69±1.81
FeSO_4_.7H_2_O	1.0	76.43±0.96
	5.0	41.47±1.37
ZnSO_4_.7H_2_O	1.0	77.34±1.33
	5.0	67.47±1.61
Al_2_(SO_4_)_3_.16H_2_O	1.0	82.94±2.63
	5.0	71.07±2.21
NaF	100	85.77±1.45
	500	75.91±2.58
NaCl	100	85.61±3.23
	500	75.23±1.36
NaBr	100	76.88±4.56
	500	75.33±3.25
NaI	100	84.69±3.45
	500	79.02±2.25

Values represent mean ± SD (n = 3) relative to untreated control sample.

### Effect of surfactants on SN4LAC activity

The effect of various surfactants at 0.1, 0.5 and 1.0 mM concentration was studied on SN4LAC activity. Enzyme was quite stable in the presence of cationic and anionic detergents. An increase in enzyme activity in the presence of CTAB (38%) and SDS (20%) was observed at 0.1 mM concentration. In the presence of non-ionic detergents, the SN4LAC was stable at lower concentrations, however, at higher concentrations [above Critical Micelle Concentration (CMC)] the SN4LAC activity decreased by 15–30% (Table 6).

**Table 6:** Effect of surfactants on SN4LAC activity.

Surfactants	Conc. (mM)	Relative activity (%)
Control	----	100
Tween 20	0.1	89.70±1.58
	0.5	78.60±2.56
	1.0	72.85±2.48
Tween 80	0.1	88.90±2.17
	0.5	76.85±1.12
	1.0	70.04±1.58
Triton X-100	0.1	100±0.58
	0.5	95.32±2.48
	1.0	84.54±2.45
SDS	0.1	120.58±1.45
	0.5	112.15±1.85
	1.0	105.80±1.78
CTAB	0.1	138.90±1.27
	0.5	133.50±0.45
	1.0	132.45±2.54

Values represent mean ± SD (n = 3) relative to untreated control sample.

## Discussion

Apart from *Streptomyces* [6], [7], [8], [9] most of the reported bacterial laccases to date are either intracellular or spore bound [5] making their industrial application unfeasible. SN4LAC is an extracellular and highly thermo-alkali-stable laccase and is thus an attractive candidate for industrial applications [12]. In this study, laccase from *B. tequilensis* SN4 (SN4LAC) has been purified and characterized. SN4LAC was purified to homogeneity with a purification fold of 28.46 and yield of 13.34%. SN4LAC is a monomeric protein with a molecular weight of 32 KDa. In contrast, the molecular weight of all other fungal [2] and bacterial laccases [5] is in the range of 50–100 KDa. This difference in molecular mass makes SN4LAC an interesting protein for studying structure-function relationship of laccases.

The UV-visible spectrum (200–800 nm) of purified SN4LAC showed characteristic peak at 600 nm corresponding to the presence of Type 1 copper center and a shoulder at 330 nm corresponding to the presence of Type 3 copper center, a characteristic of blue laccases [20] confirming it to be belonging to multicopper oxidase family. True laccase nature of SN4LAC was further supported by MALDI-TOF analysis of purified protein showed homology with laccases of other strains of *B. subtilis* being maximum with spore bound copper dependent laccase from *B.subtilis* BSn5 [19]. The extracellular laccase like protein from *Bacillus* sp. ADR did not show peak at 600 nm and was also unable to oxidize laccase specific substrates SGZ and ABTS, thus is not a true laccase [21].

SN4LAC showed wide substrate specificity. It was able to oxidize non-phenolic as well as phenolic substrates. However, the rate of oxidation of phenolic substrates varied with the nature and substitution on the phenolic ring. This difference in oxidation due to substitution on phenolic ring has been reported in case of other fungal as well as bacterial laccases [22]. It is known that laccase and tyrosinase have an overlapping range of substrates; the ability of an enzyme to oxidize SGZ and ABTS, with an inability to oxidize tyrosine, is an indicator of true laccase activity [2]. As SN4LAC was able to oxidize SGZ and ABTS but not tyrosine, it is a true laccase. The *K*m of SN4LAC towards the conventional substrates showed that binding affinity of SN4LAC was in the order of DMP>ABTS>guaiacol indicating that DMP is the most suitable substrate for SN4LAC with lowest *K*_m_ and maximum *V*_max_. Similar results have been observed for Cot A laccase from *Bacillus* sp. HR03 [23]. Further, *K*_m_ of SN4LAC for DMP is much less [8] or comparable [9] than extracellular laccases from *Streptomyces* (Table 3).

SN4LAC was also inhibited by the known inhibitors of laccase. The inhibition by sodium azide can be explained by the binding of N_3_^−^ to the trinuclear copper center, that affect internal electron transfer, which ultimately affect the overall oxidation process catalyzed by laccase [24]. EDTA and DTPA deprive the Cu^2+^ ions present at type 1 copper centre and inhibit the enzyme activity, revealing the role of Cu^2+^ ion in laccase function [25]. The enzyme activity was not observed when reducing agents such as DTT, cysteine and β-mercaptoethanol were added to the reaction mixture. This can be due to reduction of the oxidized substrate by the sulfhydryl groups of the redox reagents [26]; this has also been observed with other laccases [3].

The purified SN4LAC has temperature optima of 85°C, slightly less than the crude enzyme (90°C). The SN4LAC is completely stable for 24 h at 65°C and retained more than 80% activity at 70°C. The half life of SN4LAC was 4 h, 3 h and 1 h at 75°C, 80°C and 85°C respectively. SN4Lac is more stable than extracellular laccase from *Streptomyces*. The highest stable laccase from *S. cyaneus* CECT 3335 is having temperature optima of 70°C and retained 75% activity for 24 h only at 50°C [6]. Moreover, SN4 laccase has been found to be more thermostable in comparison to other bacterial laccases [23], [25].

Due to the difference in the redox potential of the Type 1 Copper of laccase and the substrate, the optimum pH of enzyme activity varies with the type of substrate used [1]. The optimum pH of SN4LAC for SGZ, DMP and guaiacol was observed to be 6.5, 8.0 and 8.0 respectively. The pH optima of the phenolic substrates towards alkaline range can be explained by the redox potential difference between the phenol and the T1 copper of laccase, (the driving force for electron transfer) which increases with increase in pH [4]. With ABTS, the optimum pH of SN4LAC is 5.5 which can be explained by the non-phenolic nature of ABTS [23]. The optimum pH of SN4LAC activity for various substrates is higher than that reported for other *Bacillus* spp. [22], [27]. This shift in pH to alkaline range indicates that SN4LAC is alkali-stable. This fact is supported by the pH stability experiments in which SN4LAC retained 75% activity even after 24 h incubation at pH 9.0. This stability makes it a suitable candidate to be used in industries where high pH is required.

Halides as well as metal ions are known to bind to enzymes and alter their stability [28]. In the presence of halides, SN4LAC retained 75–80% activity whereas other bacterial laccases have been reported to retain only 20–50% activity [3], [23]. Cu^2+^ and Co^2+^ increased the SN4LAC activity; similar to the results reported by Murugesan et al. [29] for laccase from *Ganoderma lucidum*. The positive effect of Cu^2+^ on laccase activity can be explained due to the filling of type 1 Copper binding site by Cu ions [1]. Fe^2+^ and Hg^2+^ inhibited the activity of SN4LAC, similar results have been reported for laccase from *Ganoderma lucidum* [29]. It has been reported that Hg^2+^ ions have strong affinity for sulfhydral (-SH) groups, binding to which causes distortion of enzyme structure [30]. Inhibitory effect of Fe^2+^ may be due to its interaction with electron transport system of laccase [29]. The enzyme was almost stable in the presence of other metal ions including Ca^2+^, Mg^2+^, Mn^2+^, Li^2+^, Zn^2+^, Ni^2+^ and Al^3+^. Stability of SN4LAC in presence of most of the metal ions makes it suitable for applications where they are present in high concentrations e.g. pulp and paper industry, wastewater containing heavy metals [1].

Ionic surfactants have been reported to inhibit laccase activity in most of the cases [3], [31]. However, the stimulation of activity by ionic surfactants has been reported for laccase from *Azospirillum lipoferum* [32]. SN4LAC activity was also stimulated by ionic surfactants i.e. CTAB and SDS. This stimulation can be explained on the basis of hypothesis given by Moore and Flurkey [33] that binding of these surfactants (generally, below the CMC) to the enzyme may cause the alterations in its enzymatic and physical characteristics.

In the presence of non-ionic detergents, the SN4LAC was stable at lower concentration, however, in contrast to ionic detergents SN4LAC activity decreased at higher concentrations of these detergents. Decrease in enzyme activity at high concentration of non-ionic detergents can be due to the reason that concentration of these detergents is higher than their CMC in the assay mixture. Difference in stability in the presence of non-ionic detergents as compared to ionic detergents at same concentration can be due to the fact that the CMC of non-ionic detergents is known to be lower by 1 order than that of ionic detergents. Thus, SN4LAC is also stable in the presence of ionic and non-ionic surfactants below their CMC. Stability of SN4LAC in the presence of surfactants makes it further useful for application in the treatment of industrial waste like dye degradation in effluent from textile industry.

## Conclusion

A novel thermo-alkali-stable extracellular laccase from *Bacillus tequilensis* SN4 has been purified to homogeneity. Purified enzyme is smaller in size than other known laccases. This makes it an interesting protein for structure-function studies. Detailed characterization showed that this laccase is highly stable at high temperature and pH. Moreover, it can work in the presence of various halides, metal ions, surfactants etc. These characteristics make it an ideal candidate for industrial applications where such extreme conditions exist.

## Supporting Information

Figure S1 **Elution profile of laccase from** ***B. tequilensis*** **SN4 from DEAE-Cellulose column.**
(DOCX)

Figure S2 **Peptide mass spectra of trypsin digested purified SN4LAC.**
(DOCX)
