# Supplementary figures and images for: Purification and Characterization of an Extracellular, Thermo-Alkali-Stable, Metal Tolerant Laccase from Bacillus tequilensis SN4
Source: PLoS One. 2014 May 28;9(5):e96951. doi: 10.1371/journal.pone.0096951 (PMC4037180; doi:10.1371/journal.pone.0096951)

**Figure S2**

**
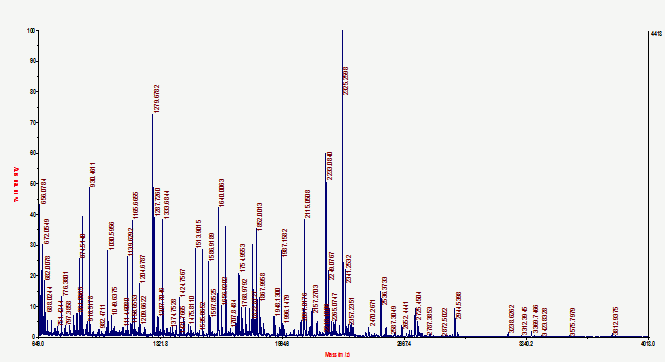
**

Supplement: Figure S2 — Peptide mass spectra of trypsin digested purified SN4LAC. (DOCX) [file pone.0096951.s002.docx]
